# Seasonal influenza vaccination in pregnant women: knowledge, attitudes, and behaviors in Italy

**DOI:** 10.1186/s12879-016-2138-2

**Published:** 2017-01-09

**Authors:** Francesco Napolitano, Paola Napolitano, Italo Francesco Angelillo

**Affiliations:** Department of Experimental Medicine, Second University of Naples, Via Luciano Armanni, 5, 80138 Naples, Italy

**Keywords:** Behaviors, Cross-sectional survey, Italy, Pregnant women, Seasonal influenza, Vaccination

## Abstract

**Background:**

The aims of this study were to assess the knowledge, attitudes, and behaviors towards seasonal influenza and its vaccination among pregnant women.

**Methods:**

A cross-sectional survey was carried out among a sample of women in the second or third trimester of pregnancy in Italy.

**Results:**

The 64.2% of the sample knew that the influenza is more dangerous for pregnant women. Women of older age, Italian, and who had a pregnancy at high-risk were more likely to have this knowledge. This knowledge was lower among women with none, primary or secondary school education. The majority of the respondents considered the vaccine not very useful during pregnancy. Those younger, unmarried, who knew that influenza is more dangerous for pregnant women, who knew that the vaccine could protect them, who reported a higher self-rated health status, and who had received information about influenza and its vaccination were more likely to have a positive attitude toward the usefulness of influenza vaccination in pregnancy. Women with secondary school education and with more than one child revealed a lower perception. Only 9.7% had received the vaccine and 21.4% of those unvaccinated would be willing to receive it. This positive attitude was higher among women with one child, who knew that the vaccine could protect them against the influenza, and who have a positive attitude toward the usefulness of the vaccination during pregnancy.

**Conclusions:**

Health educational programs are needed to improve the knowledge about seasonal influenza and vaccination rate in pregnancy.

**Electronic supplementary material:**

The online version of this article (doi:10.1186/s12879-016-2138-2) contains supplementary material, which is available to authorized users.

## Background

The seasonal influenza is one of the most frequent infections and pregnant women are at high risk with higher healthcare service use, morbidity and mortality especially in presence of chronic conditions [1–3].

The World Health Organization recommend the administration of the vaccine in healthy women who are, at the beginning of the influenza season, in the second or third trimester of pregnancy or with concomitant chronic conditions in any stage of gestation [2]. However, despite the availability of a safe and effective influenza vaccine for both the mother and the unborn [4–11], the vaccination rate in pregnant women is disappointingly low [12–15]. In Italy, the vaccination is recommended free of charge for those in the second and third trimester during the epidemic period [16].

Several studies have investigated the level of knowledge and the attitudes among pregnant women about seasonal influenza and its vaccination [17–26] and this is extremely important in order to develop strategies to improve vaccination rates. Indeed, women with higher knowledge regarding the usefulness of the vaccination are more willing to receive the vaccine during pregnancy [27–29] and the uptake is influenced by the low perception of the risks related to influenza [17, 20, 30, 31]. To date, there has been very little research concerning the level of knowledge and behaviors regarding seasonal influenza and the associated factors among pregnant women in Italy [32]. Therefore, the present survey was performed to achieve two primary objectives. The first objective was to assess the knowledge, attitudes, and behaviors towards seasonal influenza and its vaccination among pregnant women in Italy, and the second was to evaluate the determinants of knowledge and attitudes towards influenza vaccination.

## Methods

### Study setting and population

A cross-sectional survey design has been used, recruiting women in the second or third trimester of pregnancy between December 2015 and February 2016 attending Obstetrics ambulatory centers in public non-teaching hospitals in the geographic area of Naples, Italy. A two stages cluster sampling strategy has been used. Firstly, from the list of all public non-teaching hospitals in Naples, two hospitals have been randomly selected. Then, from each hospital a random sample of pregnant women has been recruited. Sample size was estimated assuming an expected positive attitude towards willingness to receive the influenza vaccine during the pregnancy of 30%, a confidence interval of 95%, and an error of 5%. The required sample was estimated in 370 pregnant women. In order to select a representative sample of the population, assuming a 10% non-response rate, the final sample size was 410 pregnant women.

### Procedure

The director of the selected hospitals received a letter requesting permission to carry out the survey, clarifying the purposes, the methods of collecting information, and the anonymity and confidentiality of the data. Eligible pregnant women in the second or third trimester of pregnancy aged 18 years or older attending the selected ambulatories were approached by one of the investigators before the clinical consultation and they were asked to complete an anonymous, face-to-face interview. Prior the interview, pregnant women were given information about the aims of the study, details about the data collection instrument, the anonymity of the interview, and that the data would be treated with respect of privacy. Signed consent form from each participant was obtained prior to data collection. Data was collected in the waiting room of the ambulatory center. Participation was voluntary and no payment or incentives has been given.

### Instrument

The face-to-face structured questionnaire [Additional file 1: Questionnaire] comprised the following five sections: (i) socio-demographic (age, educational level, marital status, number of children, employment status, medical history, self-rated health status) and pregnancy characteristics (number of pregnancies, parity, gravid, week of pregnancy); (ii) knowledge regarding influenza and its vaccination during pregnancy (risk of contract influenza, vaccination as preventive measure, vaccine recommendation, safety of the vaccine). Response options included “true,” “false,” or “do not know”; (iii) attitude towards the influenza and its vaccination during pregnancy (concern about contract influenza, usefulness of the vaccine, safety of the vaccine, intention for getting or not getting the vaccination). The attitudes were measured with a 10-point Likert scale ranging from 1 to 10; (iv) behaviors regarding influenza vaccination in previous or current pregnancy (whether or not they had received vaccination, whether or not they had received advice by the physicians to receive the vaccination). Response options included “yes” and “no”, and for each response a choice from a list of reasons; (v) sources of information about influenza and its vaccination.

A pilot study was conducted with 25 pregnant women (not included in the final sample) to determine the comprehensibility of each question. The protocol was presented and approved by the Human Research Ethical Committee of the Second University of Naples.

### Statistical analysis

The software Stata version 10.1 was used to perform all statistical analysis [33]. The first level of analysis comprised descriptive statistics of the main socio-demographic and pregnancy characteristics of the sample and of the different questions. The second level of analysis has been completed in two stages. Firstly, a bivariate analysis was carried out to assess the association between each independent variable and the different outcomes of interest using chi-square test for the categorical variables and Student’s *t*-test for the continuous variables. Secondly, multivariate analysis was performed. Multivariate logistic regression analysis was conducted to identify independent characteristics associated with the following dichotomous outcome variables: knowledge that influenza is more dangerous for pregnant women compared to non-knowledge (Model 1) and positive attitude towards willingness to receive the influenza vaccine in pregnancy compared to non-positive attitude (Model 3). A multivariate linear regression analysis was performed for the continuous outcome variable positive attitude towards the usefulness of the influenza vaccine during pregnancy (Model 2). The following characteristics of each respondent were included in all models: age (continuous, in years), highest level of education (three categories: none or primary school = 0; secondary school = 1; college degree or higher = 2), Italian nationality (no = 0; yes = 1), marital status (single/separated/divorced/widowed = 0; married = 1), number of children (none = 0; one = 1; more than one = 2), having a high-risk pregnancy (no = 0; yes = 1), and having received information about influenza and its vaccination during pregnancy (no = 0; yes = 1). Moreover, self-rated health status (continuous), knowledge that influenza is more dangerous for pregnant women than for those who are not pregnant (no = 0; yes = 1), knowledge that the vaccine could protect pregnant women against influenza (no = 0; yes = 1), and perceive dangerous for the unborn if the women contract the influenza during pregnancy (continuous) were included in Models 2 and 3. Finally, positive attitude towards the usefulness of the vaccine during pregnancy (continuous) was included in Model 3.

A stepwise procedure was used to obtain the final models according with *p* values for the variable inclusion and exclusion in the models respectively of >0.2 and <0.4. Odds ratios (ORs) and 95% confidence intervals (CIs) were calculated in the multivariate logistic regression analysis, and standardized regression coefficients (ß) were presented in the linear regression model. A two-tailed *p-*value of less than 0.05 was used to define statistical significance.

## Results

Out of the 410 pregnant women selected, 372 consented to participate for an overall response rate of 90.7%. The main socio-demographic and pregnancy characteristics of the sample are reported in Table 1. The mean age was 29 years, two thirds (67.5%) reported the secondary school as highest education, the large proportion was married, one third has at least one child, the average gestation was 27 weeks, the 12.9% had a high-risk pregnancy, and the mean self-rated health status value was 7.1.

**Table 1:** Characteristics of the sample of pregnant women

*Characteristics*	Number	Percent
*Age (years)*	29.3 ± 4.9(18-43)^a^	
*Italian Nationality*		
Yes	333	89.5
No	39	10.5
*Educational level*		
None or primary school	30	8.1
Secondary school	251	67.5
College degree or higher	91	24.4
*Marital status*		
Married	332	89.2
Other	40	10.8
*Employment status*		
Yes	175	47.1
No	197	52.9
*Number of children*		
0	176	47.3
1	124	33.3
> 1	72	19.4
*Week of pregnancy*	26.7 ± 7.4(14-39)^a^	
*Number of previous pregnancies*		
0	173	46.5
≥ 1	199	53.5
*High-risk pregnancy*		
No	324	87.1
Yes	48	12.9
*Self-rated health status*	7.1 ± 1.7(1-10)^a^	

^a^Mean ± standard deviation (range)

When it was asked about the seasonal influenza and its vaccination, 64.2% of the sample correctly answered that the influenza is more dangerous for pregnant women than for non-pregnant, 40.9% that the vaccine could protect pregnant women, one in four that vaccination was recommended during pregnancy, while only 11.2% and 7.9% of them correctly answered that it was recommended in the second or third trimester of pregnancy (Table 2). Table 3 presents the multivariate logistic and linear regression analysis of factors that remained independently associated in the models. Women of older age (OR = 1.07; 95% CI 1.01–1.13), Italian (OR = 4.97; 95% CI 1.64–15.09), and with a pregnancy at high-risk (OR = 11.43; 95% CI 2.22–58.85) were more likely to know that influenza is more dangerous for pregnant women. Moreover, this knowledge, when college degree or higher education was chosen as reference category, was lower among those with none/primary school (OR = 0.08; 95% CI = 0.02–0.36) or secondary school level of education (OR = 0.11; 95% CI = 0.04–0.28) (Model 1).

**Table 2:** Knowledge about influenza and its vaccination

	Number	Percent
The influenza is more dangerous for pregnant women than for non-pregnant women	239	64.2
The vaccine could protect pregnant women against the influenza	152	40.9
The influenza vaccine is recommended for pregnant women	89	23.9
The influenza vaccine is recommended in the second trimester of pregnancy^a^	10	11.2
The influenza vaccine is recommended in the third trimester of pregnancy^a^	7	7.9
The influenza vaccine is safe during pregnancy	40	10.7

^a^ Only for those who answered that the influenza vaccine is recommended for pregnant women

**Table 3:** Multivariate logistic and linear regression analyses indicating associations between independent variables and the different outcomes

Variable	OR	SE	95% CI	*p* value
Model 1. Knowledge that influenza is more dangerous for pregnant women (sample size = 372)				
Log likelihood = -186.11, *χ* ^2^ = 116.25 (8 df), *p* < 0.0001				
Educational level				
College degree or higher	1^a^			
None or primary school	0.08	0.06	0.02-0.36	0.001
Secondary school	0.11	0.05	0.04-0.28	<0.001
Having a high-risk pregnancy	11.43	9.55	2.22-58.85	0.004
Italian nationality	4.97	2.81	1.64-15.09	0.005
Age	1.07	0.03	1.01-1.13	0.011
Having received information about influenza and its vaccination	1.65	0.44	0.97-2.79	0.06
Number of children				
0	1^a^			
1	1.67	0.47	0.96-2.91	0.07
Marital status	0.63	0.28	0.26-1.53	0.313
Variable	Coeff.	SE	*t*	*p* value
Model 2. Positive attitude towards the usefulness of the influenza vaccine in pregnancy (sample size = 372)				
*F* (12, 359) = 14.54, *p* < 0.0001, *R* ^2^ = 0.32%, adjusted *R* ^2^ = 0.3%				
Age	-0.09	0.02	-3.54	<0.001
Having received information about influenza and its vaccination	1.06	0.23	4.57	<0.001
Knowledge that influenza is more dangerous for pregnant women	0.66	0.29	2.23	<0.001
Knowledge that the vaccine could protect pregnant women against influenza	1.21	0.29	4.18	<0.001
Educational level				
College degree or higher	1^a^			
None or primary school	0.91	0.58	1.58	0.115
Secondary school	-0.85	0.29	-2.89	0.004
Number of children				
0	1^a^			
> 1	-0.82	0.32	-2.58	0.01
Marital status	-1.03	0.41	-2.56	0.011
Self-rated health status	0.15	0.07	2.15	0.033
Having a high-risk pregnancy	0.74	0.38	1.94	0.053
Perceive dangerous for the unborn if the women contract the influenza during pregnancy	0.06	0.05	1.05	0.295
Italian nationality	0.42	0.46	0.89	0.375
Variable	OR	SE	95% CI	*p* value
Model 3. Positive attitude towards willingness to receive the influenza vaccine in pregnancy (sample size = 336)				
Log likelihood = -150.08, *χ* ^2^ = 48.99 (11 df), *p* < 0.0001				
Positive attitude toward the usefulness of influenza vaccination in pregnancy	1.35	0.11	1.14-1.59	<0.001
Knowledge that the vaccine could protect pregnant women against influenza	3.03	1.24	1.35-6.77	0.007
Number of children				
0	1^a^			
1	2.41	0.92	1.15-5.08	0.02
> 1	3.34	1.62	1.29-8.67	0.013
Knowledge that influenza is more dangerous for pregnant women	0.45	0.19	0.19-1.06	0.068
Italian nationality	2.58	1.59	0.77-8.62	0.122
Perceive dangerous for the unborn if the women contract the influenza during pregnancy	0.89	0.07	0.76-1.05	0.183
Self-rated health status	0.88	0.08	0.72-1.06	0.185
Educational level				
College degree or higher	1^a^			
Secondary school	1.44	0.52	0.71-2.92	0.306
Marital status	0.6	0.3	0.22-1.65	0.326
Age	0.97	0.03	0.91-1.03	0.364

^a^Reference category

With regard to attitudes, women feel dangerous for them and for the unborn to contract the influenza during pregnancy with a mean value respectively of 7.1 and 7.3, out of a maximum score of 10. Despite these perceptions, the majority considered the influenza vaccine during pregnancy not very useful with an average value of 4.9, and feels the vaccine potentially dangerous for their health (mean 6.7) or for the unborn (mean 7). Multivariate linear regression analysis showed that those younger, unmarried, who knew that influenza is more dangerous for pregnant women, who knew that the vaccine could protect pregnant women, who reported a higher self-rated health status, and who had received information about influenza and its vaccination were more likely to have a positive attitude toward the usefulness of influenza vaccination during pregnancy. Moreover, women with secondary school level of education, compared to those with a college degree or higher education, and those who have more than one child, compared to those with no children, revealed a lower perception towards the usefulness of vaccination during pregnancy (Model 2).

Only 9.7% of the women had received influenza vaccination and the most important reason for being vaccinated was the advice of the physician (88.9%), whereas, for those unvaccinated the reasons most often mentioned were that they had not received information by physicians (34.9%), fear that the vaccine would be harmful (32.2%), and believed that the vaccine was not needed (26.7%).

Among unvaccinated women, only 21.4% reported a positive intention regarding vaccination during pregnancy. Multivariate logistic regression analysis revealed that this positive attitude was significantly higher among those who had one (OR = 2.41; 95% CI = 1.15–5.08) or more than one child (OR = 3.34; 95% CI = 1.29–8.67) compared to those with no children, who knew that the vaccine could protect pregnant women (OR = 3.03; 95% CI = 1.35-6.77), and who have a positive attitude toward the usefulness of the vaccination during pregnancy (OR = 1.35; 95% CI = 1.14–1.59) (Model 3). The most frequently selected reasons for the positive attitude were the recommendation by the physician (59.7%), that the influenza could be harmful for them (15.9%) and for the unborn baby (19.4%). Reasons for unwilling to receive the vaccine included concerns for the unborn (65.4%) or for the pregnant woman (63.1%).

During their current pregnancy, only 40.3% had received information about influenza and its vaccination mainly by physicians (62%), internet (32.1%), and mass media (6.6%). Moreover, the majority would like to learn more about influenza (59.9%) and its vaccination (61.6%).

## Discussion

So far as we know, this is the first time that has been examined the level of knowledge, the awareness, and the behavior about seasonal influenza and its vaccination and also the associated factors in a large group of pregnant women in Italy.

It is important to consider that comparison with similar studies may be partly difficult due to differences regarding the study design, the study period, the strategies of the data collection, the different characteristics of the populations sampled, and the public health preventive activities. One of the main findings from this study is that the overall knowledge and the vaccination uptake were relatively poor. Indeed, less than two-thirds answered that the influenza is more dangerous for pregnant women than for non-pregnant, less than half that the vaccine could protect pregnant women, and one in four that the vaccination is recommended during pregnancy. When compared to other studies, the observed knowledge was higher. For example, in India none knew about influenza vaccination requirement during pregnancy [34], in Canada 36% knew that influenza was more severe in pregnant women [35], in the United States 51% identified that seasonal flu is more dangerous for pregnant women and 54% that the seasonal flu shot is safe in pregnancy [17]. Similarly, in Australia 23% of women believed that the vaccination during pregnancy was not safe and 30% that it would not protect them [19]. A considerably higher level of knowledge has been observed in the United States with 77% pregnant women who were aware that the vaccine is recommended [23] and in Pakistan 75% acknowledged that the vaccine is safe for pregnant women [24].

The present survey revealed a very low overall vaccination rate with a value of 9.7% and this of particular concern. In theory, one would expect that this group have a higher rate because of a better understanding of the need to be vaccinated. However, this is not observed in this sample. Therefore, it is important to design and to implement interventions in order to increase the vaccination rate. The value observed in the present study is higher than those reported in other countries, since no women had received influenza vaccination in the already mentioned study in India [34], 3% in Turkey [36], 4% in Thailand [26], and it is almost identical to the 6% in Iran [37], 10.9% in Germany [38], and 16% in Canada [39]. Whereas, it is substantially below the levels observed in Australia with an uptake of 27% [20], in France of 39% [40], in Belgium of 42.8% [41], and in the United States with levels ranging from 35% [14] to 66.4% [42]. One of the most commonly provided reasons by the unvaccinated was that their doctor had not discussed during their pregnancy. The finding that more than one third of those unvaccinated did not receive a health care provider recommendation is in line with previous studies [34, 36, 41, 43, 44]. Another commonly provided reason was the concern that vaccination may not be safe during pregnancy. Despite the extensive research demonstrating vaccine safety, this is alarming and this is accordance with previous studies [14, 17, 20, 40, 43, 45]. This stressed the role of the physicians. Indeed, physicians are an important channel for reaching pregnant women and for delivering key messages in order to contribute to improve the acceptance of the vaccine. The finding regarding the sources of information about influenza, which might improve the level of knowledge and acceptance rate, showed that physicians were the deeply trusted source and this has been observed in previous studies [17, 21, 23, 36, 46]. Of the unvaccinated women, one-fifth indicated that they intended to receive the vaccination during pregnancy. This value is almost identical to the 21.6% found in the United States among pregnant minority women [47]. By contrast, higher values have been observed in the already mentioned survey in Thailand with 42% of the women who reported being willing to receive the seasonal influenza vaccine [26], in the United States 82.8% said they would be immunized if recommended by their physician [23], and in Pakistan 87% were willing to accept the vaccine [24].

Multivariate regression models have been developed for estimating the association of individual characteristics and specific outcomes of interest. Several socio-demographic characteristics, including age, educational level, and marital status, resulted significantly associated with greater knowledge and positive attitude. Women of older age had higher level of knowledge and, not surprisingly, those with higher education level were more likely to know that the influenza is dangerous for pregnant women and to report a positive attitude towards the utility of the vaccination during pregnancy. Prior study has shown similar findings [38]. The fact that women with a high-risk pregnancy were more likely to know that the influenza is dangerous for pregnant can serve as a focus when promoting health education interventions. Moreover, an association has been identified between knowledge concerning influenza and vaccination and the attitude toward the usefulness of the vaccination during pregnancy. Women with a better knowledge had a significantly higher likelihood of having a positive attitude toward the usefulness of the vaccination during pregnancy. Adequate knowledge and awareness of a disease are the key prerequisites for its prevention and control, given that adequate knowledge is a basis for adopting appropriate attitudes and practices.

Care is required in interpreting the findings of this study for potential limitations typical of population-based questionnaire surveys. Firstly, it is difficult, as in the cross-sectional studies, to precisely determine temporal sequence between the dependent and independent variables. Secondly, there was a possibility of bias due to participants with favorable attitudes towards vaccination potentially being more likely to respond to the questionnaire. This suggests that responses can be biased due to forgetfulness or exaggeration of attitude and behaviors, social desirability, or affected by feelings at the time of the interview. Thirdly, the data were obtained from interviews and the answers were not verified through chart review and, therefore, it is not possible to be certain that the respondents answered correctly and recall bias could have occurred. Finally, a non-standardized questionnaire has been used, although this limitation has been partially solved by piloting the questions on a group of pregnant women. Despite the mentioned limitations, the major strength of the study resides in the high response rate, making the results robust enough to be representative of the population.

## Conclusions

In conclusion, the low rate of vaccination and of women who have expressed a positive attitude towards to receive the vaccine support the need to develop health educational programs in order to improve the level of knowledge about seasonal influenza and its vaccination in this population focusing on the efficacy and safety. Gynecologists and primary care physicians, who are the providers of health care during pregnancy, with their consultations and recommendations should play a more substantial role in promoting this vaccination and to increase the uptake in pregnant women.

## Additional file

Additional file 1: Questionnaire. (DOCX 48 kb)

## Abbreviations

CI: Confidence interval
OR: Odds ratio
