# Supplementary material for: Seasonal influenza vaccination in pregnant women: knowledge, attitudes, and behaviors in Italy
Source: BMC Infect Dis. 2017 Jan 9;17:48. doi: 10.1186/s12879-016-2138-2 (PMC5223411; doi:10.1186/s12879-016-2138-2)
Supplement: Additional file 1: — Questionnaire. (DOCX 48 kb) [file 12879_2016_2138_MOESM1_ESM.docx]

***Additional file 1***

**QUESTIONNAIRE**

**Respondent n° _________ Date ___________** **Ambulatory center ____________________**

**Section A.**

**I will ask you some questions to gather information about your socio-demographic and clinical characteristics.**

**A1.** How old were you on your last birthday? ______________

**A2.** What is your nationality? □ Italian □ Other **________________**

**A3.** What is your marital status? □ Married □ Single (never married) □ Other ___________

**A4.** What is your highest educational level? ____________________________________________

**A5.** What is your occupation?___________________________________________

**A6.** What is the current occupation of your husband/partner?________________________________________

**A7.** How many sons do you have? ______

**A8.** How many pregnancies have you had? ________

**A9.** What is your week of pregnancy _____

**A10.** Do you have a high-risk pregnancy? □ No □ Yes

**A11.** How would you rate your health status on a 1 to 10 scale with 1 meaning bad at all and 10 very good health status??

**bad**  1 2 3 4 5 6 7 8 9 10 **very good**

**Section B.**

I will ask you some questions to explore your knowledge related seasonal influenza and its vaccination.

|  | **Yes** | **No** | **Do not know** |
| --- | --- | --- | --- |
| The influenza is more dangerous for pregnant women | □ | □ | □ |
| The vaccine could protect pregnant women against the influenza | □ | □ | □ |
| The vaccination is recommended for pregnant women | □ | □ | □ |
| The vaccination is recommended in the first trimester of pregnancy | □ | □ | □ |
| The vaccination is recommended in the second trimester of pregnancy | □ | □ | □ |
| The vaccination is recommended in the third trimester of pregnancy | □ | □ | □ |
| The vaccination is safe in pregnancy | □ | □ | □ |

**Section C.**

**I would like to know your attitudes towards influenza vaccination in pregnancy. Answer the following questions as truthfully as possible.**

**C1.** How do you perceive is dangerous for you to contract the influenza during pregnancy? (1 not dangerous; 10 very dangerous)

not dangerous 1 2 3 4 5 6 7 8 9 10 very dangerous

**C2.** How do you perceive is dangerous for your unborn if you contract the influenza during pregnancy? (1 not dangerous; 10 very dangerous)

not dangerous 1 2 3 4 5 6 7 8 9 10 very dangerous

**C3.** How would you rate the utility of the influenza vaccine during pregnancy? (1 useless; 10 very useful)

useless 1 2 3 4 5 6 7 8 9 10 very useful

**C4.** How do you perceive that is dangerous for you to get the influenza vaccination during pregnancy? (1 not dangerous; 10 very dangerous)

not dangerous 1 2 3 4 5 6 7 8 9 10 very dangerous

**C5.** How do you perceive that is dangerous for your unborn if you get the influenza vaccination during pregnancy? (1 not dangerous; 10 very dangerous)

not dangerous 1 2 3 4 5 6 7 8 9 10 very dangerous

**Section D.**

**I am going to ask you some questions to know the sources and need of information about influenza vaccination in pregnancy.**

(**D1** is only for those who have had at least one previous pregnancy)

**D1.** In your previous pregnancies have you been vaccinated against influenza?

First pregnancy □ no □ yes Second pregnancy □ no □ yes Third pregnancy □ no □ yes

**D2.** Have you received the influenza vaccine during this pregnancy?

□ No, why? _____________________________________________

□ Yes, why? ______________________________________________ **(go to D4)**

**D3.** Do you intend to receive the influenza vaccine?

no yes

why? why?

**(more than one answer is allowed) (more than one answer is allowed)**

□ I do not feel at risk □ I feel at risk

□ The vaccine could be harmful for my health □ The influenza could be harmful for my health

□ The vaccine could be harmful for my unborn □ The influenza could be harmful for my unborn

□ The vaccine is not useful □ The vaccine is useful

□ The physician did not recommend the vaccination □ The physician recommended the vaccination

□ Other ______________________ □ Other ____________________

**D4.** Did the primary care physician and/or specialist(s) recommend you the influenza vaccine?

□ No □ Yes, which physician/s? ____________________

**Section E.**

**I am going to ask you some questions to know the sources and need of information about influenza vaccination in pregnancy**

**E1.** Do you receive information about influenza during pregnancy?

□ No □ Yes, if the answer is yes, ask: From which of the following sources (more than one answer is allowed) □ Physicians □ Mass media □ Friends □ Family members □ Internet □ Other (specify ________)

**E2.** Do you receive information about influenza vaccination during pregnancy?

□ No □ Yes, if the answer is yes, ask: From which of the following sources (more than one answer is allowed) □ Physicians □ Mass media □ Friends □ Family members □ Internet □ Other (specify ________)

**E3.** Do you feel you need more information about influenza during pregnancy? □ No □ Yes

**E4.** Do you feel you need more information about influenza vaccination during pregnancy? □ No □ Yes
